# Microbiota-derived I3A protects the intestine against radiation injury by activating AhR/IL-10/Wnt signaling and enhancing the abundance of probiotics

**DOI:** 10.1080/19490976.2024.2347722

**Published:** 2024-05-05

**Authors:** Li-Wei Xie, Shang Cai, Hai-Yan Lu, Feng-Ling Tang, Rui-Qiu Zhu, Ye Tian, Ming Li

**Affiliations:** a Suzhou Key Laboratory for Radiation Oncology, Department of Radiotherapy and Oncology, The Second Affiliated Hospital of Soochow University, Suzhou, China; b State Key Laboratory of Radiation Medicine and Protection, School of Radiation Medicine and Protection, Medical College of Soochow University; Collaborative Innovation Center of Radiation Medicine of Jiangsu Higher Education Institutions, Soochow University, Suzhou, China

**Keywords:** Radiotherapy, gastrointestinal tract toxicity, gut microbiota, tryptophan metabolites, indole-3-carboxaldehyde, intestinal stem cells

## Abstract

The intestine is prone to radiation damage in patients undergoing radiotherapy for pelvic tumors. However, there are currently no effective drugs available for the prevention or treatment of radiation-induced enteropathy (RIE). In this study, we aimed at investigating the impact of indole-3-carboxaldehyde (I3A) derived from the intestinal microbiota on RIE. Intestinal organoids were isolated and cultivated for screening radioprotective tryptophan metabolites. A RIE model was established using 13 Gy whole-abdominal irradiation in male C57BL/6J mice. After oral administration of I3A, its radioprotective ability was assessed through the observation of survival rates, clinical scores, and pathological analysis. Intestinal stem cell survival and changes in the intestinal barrier were observed through immunofluorescence and immunohistochemistry. Subsequently, the radioprotective mechanisms of I3A was investigated through 16S rRNA and transcriptome sequencing, respectively. Finally, human colon cancer cells and organoids were cultured to assess the influence of I3A on tumor radiotherapy. I3A exhibited the most potent radioprotective effect on intestinal organoids. Oral administration of I3A treatment significantly increased the survival rate in irradiated mice, improved clinical and histological scores, mitigated mucosal damage, enhanced the proliferation and differentiation of Lgr5^+^ intestinal stem cells, and maintained intestinal barrier integrity. Furthermore, I3A enhanced the abundance of probiotics, and activated the AhR/IL-10/Wnt signaling pathway to promote intestinal epithelial proliferation. As a crucial tryptophan metabolite, I3A promotes intestinal epithelial cell proliferation through the AhR/IL-10/Wnt signaling pathway and upregulates the abundance of probiotics to treat RIE. Microbiota-derived I3A demonstrates potential clinical application value for the treatment of RIE.

## Introduction

Radiotherapy is a crucial approach for treating malignant tumors. Despite continuous advancements in precise radiation techniques, in the context of abdominopelvic tumor radiotherapy, unavoidable anatomical factors lead to the irradiation of intestinal tissues. This results in a high occurrence rate of acute radiation enteropathy (up to 80%) among patients, with approximately 50% progressing to chronic damage.^1,2^ Radiation-induced enteropathy (RIE) not only restricts the escalation of radiation dosage and therapeutic efficacy, but also impacts patients’ quality of life. RIE primarily manifests with symptoms such as diarrhea, varying degrees of abdominal pain, rectal bleeding, weight loss, and intestinal obstruction.^3^ Currently, clinical interventions for RIE involve medication, probiotic treatments, and surgical procedures. However, these approaches have not yielded satisfactory results. For instance, the success rate of small bowel transplantation (approximately 60%) is notably lower than that of other organ transplants.^4^ Therefore, even in the current era where advanced radiotherapy techniques have been standardized, research on therapeutic strategies for RIE remains clinically significant for patients with abdominopelvic tumors undergoing radiotherapy.

The disruption of intestinal mucosal barrier function is one of the main mechanisms underlying RIE.^5^ Increasing evidence suggests that the gut microbiota play a role in regulating intestinal mucosal barrier function and the renewal of intestinal stem cells (ISCs), making them potential therapeutic targets for RIE. Several preclinical studies have discovered that probiotics and fecal microbiota transplantation can prevent and treat acute RIE. ^6–8^ However, the therapeutic effect of probiotics in the treatment of RIE is inconsistent, mainly due to factors such as dietary and age variations, antibiotic use, tumor presence, and intestinal disorders.^9^ Within the intestines, food undergoes fermentation by the gut microbiota, resulting in a diverse array of metabolites.^10^ By leveraging these metabolites, the gut microbiota can directly or indirectly regulate the functions of both local and distant organs in the host, thus influencing the pathophysiological processes of hosts. Currently, short-chain fatty acids (SCFAs), aromatic amino acids (AAAs), bile acids(BAs), and others have been identified as signaling molecules in the microbiota-host dialog, which participate in the regulation of host physiological functions under radiation conditions.^11,12^ Butyrate salts regulate genes associated with epithelial barrier function, thus promoting intestinal barrier function and aiding in the repair of RIE.^13^ Administering valeric acid, a metabolite generated by the gut microbiota, improves survival rates in irradiated mice and preserves gastrointestinal function.^14^ Therefore, identifying key metabolic products regulated by gut microbiota can help address the current challenge of inconsistent efficacy in probiotic therapies.

Microbiota-dependent tryptophan (Trp) degradation products are extensively produced in the gastrointestinal tract and have profound impacts on host physiology, including the maintenance of epithelial barrier function and immune homeostasis.^15,16^ Trp metabolism in the gut primarily occurs through three major pathways: the indole pathway mediated by the gut microbiota, and the kynurenine and serotonin pathways mediated by the host.^15^ The indole pathway in the gut includes the direct transformation of Trp by intestinal microorganisms into several molecules, such as indole and its derivatives. Indole derivatives mainly include indole-3-acetic acid (IAA), indole-3-propionic acid (IPA), indole-3-acetaldehyde (IAAld), indole-3-carboxaldehyde (I3A), and indoleacrylic acid (ILA).^16^ Research suggests that gut microbiota-derived Trp metabolites can not only serve as biomarkers for radiation damage, but also hold potential as radioprotective agents.^17,18^ In this study, we examined the radioprotective capacity of Trp metabolites driven by the gut microbiota at the level of intestinal organoids and found that I3A had the best radioprotective effect. I3A has been reported to extend the lifespan of mice after high dose ionizing radiation (IR) exposure.^19^ In addition to microbial production, I3A can be produced by bacterial metabolism in vegetables, making it widely present in cruciferous vegetables such as cabbage and broccoli. As a component of vegetable extracts, I3A exhibits various biological effects, including anti-inflammatory, anti-infective, anti-aging, and analgesic properties.^20^ I3A is recognized as a ligand for the aryl hydrocarbon receptor (AhR), a ligand-activated transcription factor that was initially identified for its role in xenobiotic binding and metabolism; however, it has now been associated with various activities, such as immune regulation, maintenance of intestinal barrier homeostasis, and microbial symbiosis.^21^ In inflammatory bowel disease models, AhR gene knockout mice exhibit highly severe intestinal mucosal barrier disruption and higher mortality rates than wild-type mice.^22^ Furthermore, a phase III randomized controlled clinical study confirmed the efficacy of AhR activators for treating inflammatory bowel disease.^23^ Furthermore, recent research has found that I3A promotes hematopoietic stem cells to ameliorate IR-induced hematopoietic injury.^24^ However, it is not clear whether I3A can exert intestinal radioprotective effects in RIE mouse models, and the related potential mechanisms of action need further elucidation.

In this study, we aimed to investigate whether I3A can ameliorate RIE in a mouse model. Our observations indicate that oral administration of I3A can alleviate radiation-induced intestinal damage and loss of ISCs. Mechanistically, IR promoted I3A expression depending on the gut microbiota, and I3A administration improved the composition of the gut bacterial community. Importantly, the AhR/IL-10/Wnt3 axis was found to be essential for the radioprotective effects of I3A. Overall, our findings provide novel insights into the functionality and potential protective mechanisms of microbiota-derived metabolites in the context of RIE for preclinical research.

## Results

### I3A protects intestinal crypt organoids and HIEC-6 cells against radiation-induced injury

**Figure 1.**
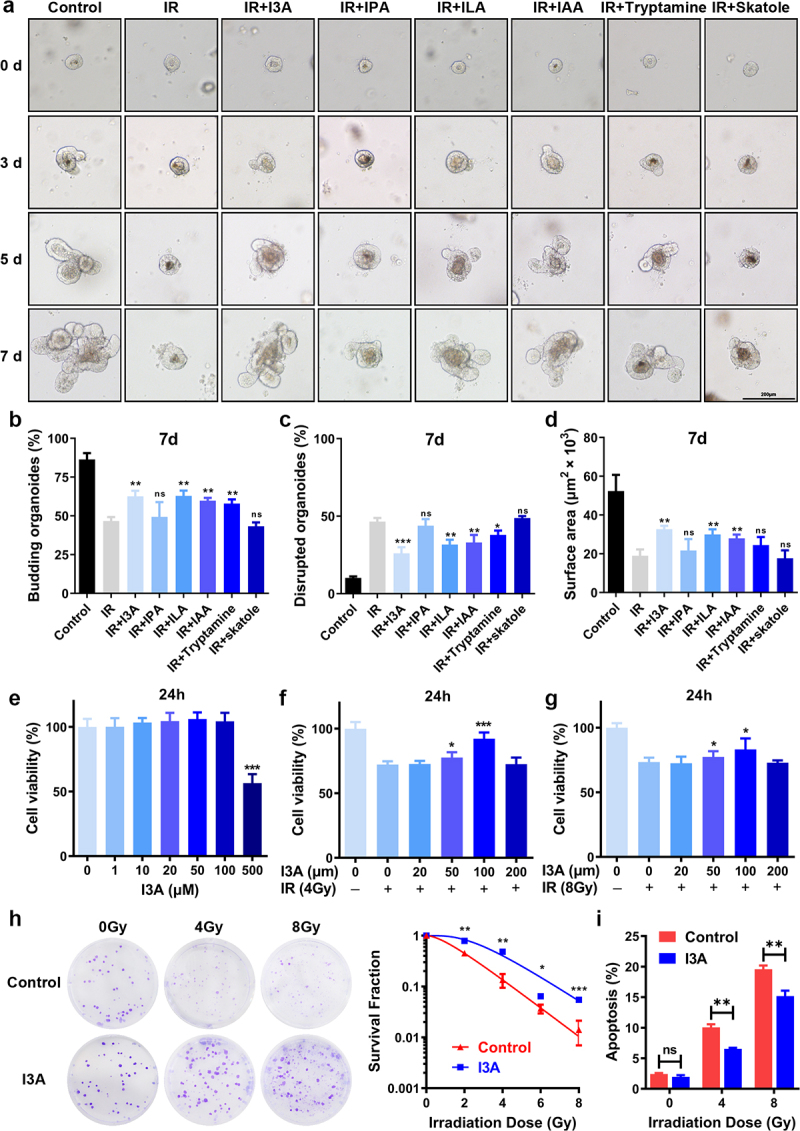
I3A protects intestinal crypt organoids and HIEC-6 against radiation-induced injury. (a) Representative phase contrast images of intestinal organoids cultured with or without tryptophan metabolites at 0, 3, 5 or 7 d post-IR (scale bar = 200 μm). (b) The budding/total organoids (%) at 7 d post-IR. At least 50 intestinal organoids were counted in each group. (c) The number of damaged organoids per well at 7 d post-IR. At least 50 intestinal organoids were counted in each group. (d) Surface area per organoid at 7 d post-IR. At least 50 intestinal organoids were counted in each group. (e) Cytotoxicity of I3A in HIEC-6 cells. HIEC-6 cells were treated with the indicated concentrations of I3A for 24 h. Cell viability was determined using the CCK-8 assay. (f-g) Effects of I3A on cell viability post IR in HIEC-6 cells. HIEC-6 cells were pretreated with the indicated concentrations of I3A 1 h before 4 or 8 Gy IR, and cell viability was measured after 24 h. (h) Clonogenic survival assay of HIEC-6 cells pretreated with 100 μM I3A or vehicle 1 h following 0, 2, 4, 6, or 8 Gy radiation. (i) Effects of I3A on cell apoptosis post IR in HIEC-6 cells. Cell apoptosis was measured using Annexin V/PI staining in HIEC-6 cells treated with 100 μM I3A or vehicle 1 h before 4 or 8 Gy irradiation. Data are presented as the mean ± S.D. of three independent experiments. *p < .05; **p < .01; ***p < .001.

To screen the most effective indole derivatives for protection against radiation, primary indole derivatives including IAA, IPA, I3A, ILA, 3-methylindole (skatole) and tryptamine were tested at the level of mouse intestinal organoids. I3A was found to significantly improve the rate of budding organoids, disrupted organoids and the surface area of intestinal organoids compared to the control (Figure 1a–d), indicating that I3A had protective effects on intestinal organoids and promoted the survival of ISCs. Although metabolites such as ILA and IAA have a certain protective effect, I3A has the strongest radioprotective effect from the comprehensive effect.

I3A facilitated the proliferation of irradiated HIEC-6 cells in a dose-dependent manner. I3A treatment at concentrations lower than 100 μM did not show a cytotoxic effect on cell proliferation in HIEC-6 cells, and the radioprotective effect was maximum for 100 μM I3A (Figure 1e–g). Consistent with the results of the CCK-8 assay, the LDH assay also revealed that 100 μM I3A exhibited the most significant radioprotective effect (Figure S1). Colony formation assays revealed an increase in the survival and clonogenic potential of irradiated HIEC-6 cells upon I3A treatment, especially in the 4 Gy and 8 Gy dose groups, compared to the control group (Figure 1h). Propidium iodide (PI) staining revealed a significant increase in cell death in HIEC-6 cells after 4 Gy and 8 Gy X-ray irradiation compared to that in sham-irradiated HIEC-6 cells. In contrast, I3A treatment effectively reduced radiation-induced cell death in HIEC-6 cells (Figure 1i). Thus, these results implied that I3A administration protects against radiation-induced cell injury *in vitro*.

### Oral gavage of I3A ameliorates total abdominal irradiation-associated intestinal injury

**Figure 2.**
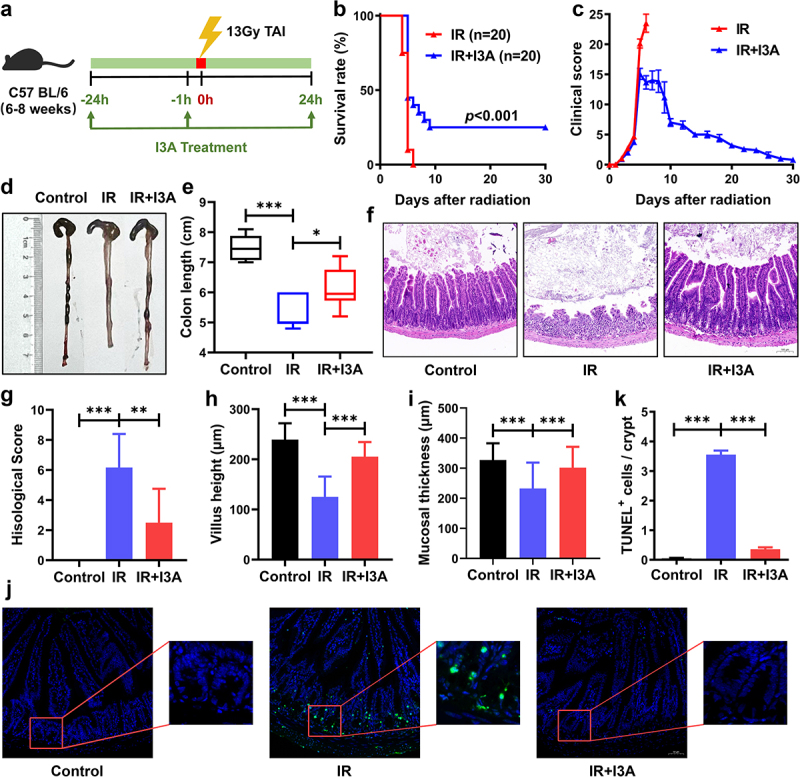
Oral gavage of I3A ameliorates TAI-associated intestinal injury. (a) Schematic diagram of I3A treatment. I3A (200 mg/kg) was administered by oral gavage into SPF C57BL/6J mice in three doses: 1 d before radiation, 1 h before radiation, and 1 d after radiation. Then the mice were received 13 Gy TAI. (b) Kaplan‒Meier analysis of C57BL/6J mice treated with I3A or vehicle following 13 Gy TAI (n = 20/group). *p* < .001 by log-rank test between IR and IR+I3A groups. (c) Clinical scores were determined as described in the materials and methods. (d-e) Colon length of mice in each group. The colon tissues were obtained at 3 d post-TAI. (f) The morphology of the small intestine was shown by H&E staining (scale bar = 100 μm). The small intestine tissues were obtained at 3 d post-TAI. (g) Histopathological scoring was performed as described in the materials and methods. (h) Villus height and (i) mucosal thickness of small intestines in each group. (j) Representative TUNEL-stained intestinal sections of various groups (scale bar = 50 μm). (k) Number of TUNEL^+^ cells per crypt at 6 h post-TAI. Data are presented as the mean ± S.D., *p < .05; **p < .01; ***p < .001; n = 6/group.

First, we identified whether I3A replenishment could protect against death from exposure to irradiation. After exposure to 13 Gy total abdominal irradiation (TAI), the animal survival rate was increased by 25% in the I3A group (Figure 2b). The clinical score was significantly lower in I3A-treated mice (Figure 2c). I3A significantly attenuated colon length shortening in mice at 3 d post irradiation (Figure 2d,e). Histological and pathological analysis suggested that I3A significantly reduced injury scores (Figure 2f,g), restored the shortening of intestinal villi and mucosa caused by radiation (Figure 2h, i), reduced the upregulation of terminal deoxynucleotidyl transferase dUTP nick end labeling (TUNEL)-positive cells in irradiated mice (Figure 2j,k). Thus, we conclude that I3A administration prevents radiation-induced gastrointestinal injury *in vivo*.

### I3A promotes the proliferation and differentiation of crypt cells and maintains intestinal barrier integrity after TAI

**Figure 3.**
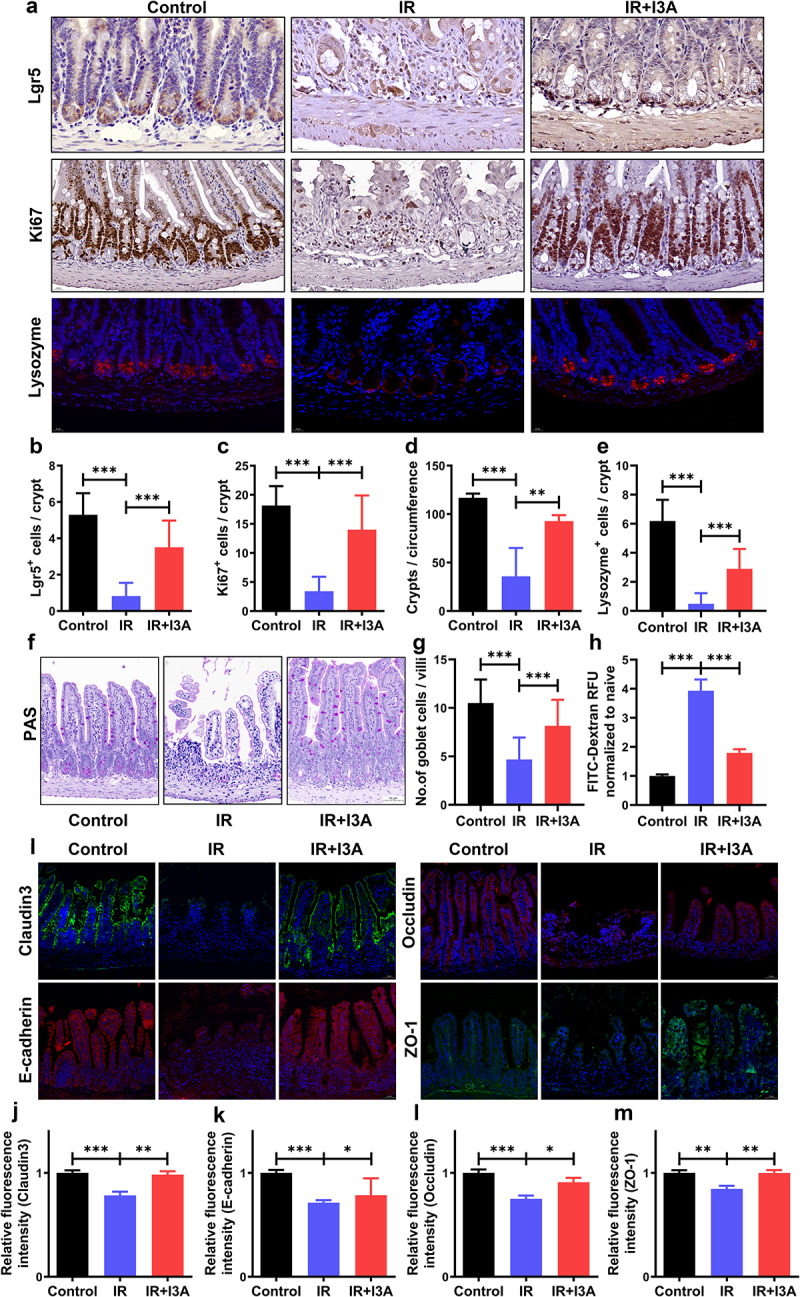
I3A promotes the proliferation and differentiation of crypt cells and maintains intestinal barrier integrity after TAI. (a) Representative images of Ki67 and Lgr5 IHC (in dark brown) and lysozyme IF (in red) staining of the vehicle- and I3A-treated mice at 3 d after TAI (scale bar = 20 μm). (b) Histogram showing the number of Lgr5^+^ ISCs per crypt. Lgr5^+^ ISCs were quantified in at least 30 crypts per mouse. (c-d) Histograms showing the number of Ki67^+^ cells per crypt and surviving crypts per circumference determined from panel a. At least 30 well-oriented crypts per mouse were counted. (e) The number of lysozyme^+^ cells in each crypt was detected. (f) Representative PAS-stained intestinal sections of various groups (scale bar = 50 μm). Histological staining by PAS was performed to show goblet cells per villus. (g) Number of PAS^+^ cells per villus at 3 d post-TAI. (h) Relative FITC-dextran level in the serum 3 d post-TAI. (i) Representative IF images of the small intestinal sections showing the expression of Claudin-3, E-cadherin, Occludin and ZO-1 at 3 d post-TAI (scale bar = 50 μm). (j-m) Relative fluorescence intensities of Claudin 3, E-cadherin, Occludin, and ZO-1, as determined from panel i (n = 6/group). Data are presented as the mean ± S.D., *p < .05; **p < .01; ***p < .001; n = 6/group.

To assess whether I3A affects the proliferation of crypt epithelial cells, intestinal sections were examined by immunohistochemistry (IHC) using antibodies against Lgr5 and Ki67. Of these, Lgr5 is a widely recognized marker for active ISCs, which is crucial for intestinal regeneration after radiation.^25^ The number of Lgr5^+^ ISCs per crypt was partially recovered after treatment with I3A (Figure 3a,b). Ki67 serves as a proliferation marker, facilitating the assessment of cell proliferation rates.^26^ I3A increased the number of Ki67^+^ cells per crypt and the average number of Ki67^+^ crypts per circumference (Figure 3a,c,d). We further investigated whether I3A affected the differentiation of ISCs into paneth cells and goblet cells. Lysozymes, produced by Paneth cells, play a vital role in the antimicrobial functions of the intestinal epithelium, contributing to intestinal homeostasis.^27^ In this study, the counts of lysozyme^+^ paneth cells and PAS^+^ goblet cells were reduced after TAI, but these changes were attenuated by I3A treatment (Figure 3a,e–g).

The intestinal epithelial barrier of normal mice prevented fluorescein isothiocyanate (FITC)-dextran from entering the blood, so intestinal permeability was measured by orally administering FITC-dextran to mice. I3A treatment significantly reduced the levels of FITC-dextran in the serum compared to the corresponding levels in the serum of vehicle-treated mice (Figure 3h). Tight junctions (TJs) play a crucial role in maintaining the integrity and functionality of the intestinal barrier. They comprise proteins such as occludin and claudins and are intricately linked to the cytoskeleton through cytoplasmic plaque proteins, namely zonula occludens protein-1 and −2 (ZO-1 and ZO-2).^28,29^ Specific claudins, such as Claudin-3, play a pivotal role in forming the paracellular barrier; there is evidence that increasing the Claudin-3 levels contributes to colitis restoration.^30^ To deepen our understanding of TJ dynamics in the context of intestinal barrier function in response to I3A treatment, we investigated the expression of TJ proteins through immunofluorescence (IF) analysis. The I3A treatment alleviated the destruction of TJ protein expression, such as Claudin3, Occludin, E-cadherin and ZO-1 (Figure 3i–m). Collectively, these results demonstrate that I3A therapy preserves the normal architecture of the small intestinal crypt-villus structure, stimulates ISCs proliferation, and promotes ISCs differentiation into paneth cells and goblet cells, thereby maintaining the integrity of the intestinal mucosal barrier.

### Gut microbiota-derived I3A changes the irradiation-induced intestinal bacterial structure

**Figure 4.**
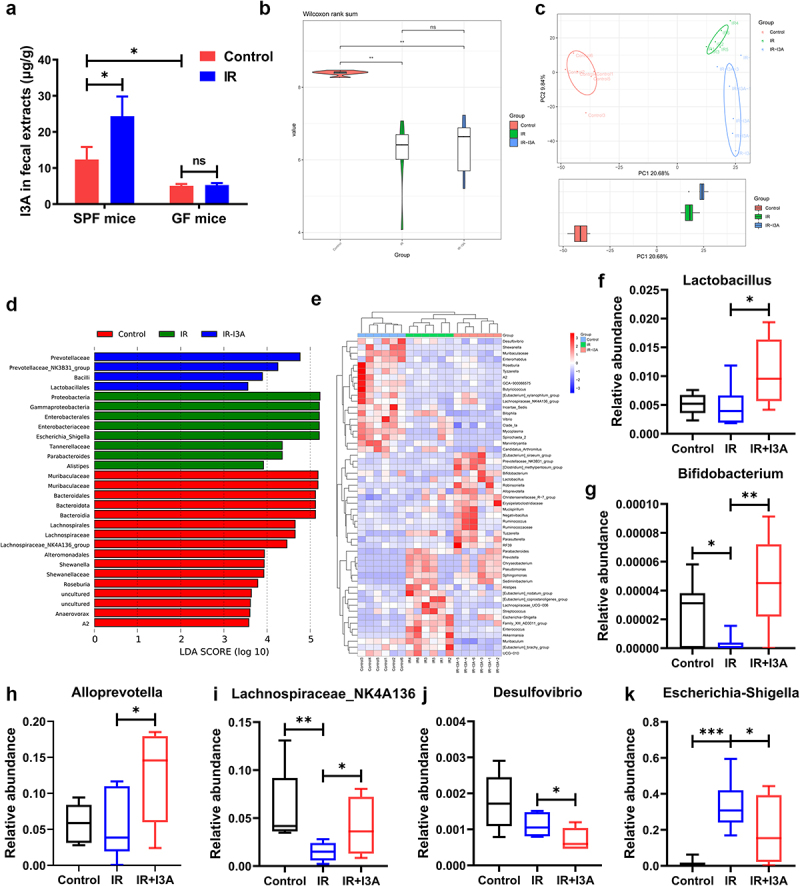
I3A treatment affects the bacterial composition of the gut microbiota. (a) The concentration of I3A in the fecal pellets of SPF and GF C57BL/6J mice was measured at 0 or 3 d after 13 Gy TAI. (b) Wilcoxon plot of the microbial alpha diversity in each group at 3 d after 13 Gy TAI. (c) Principal coordinate analysis (PCoA) of gut microbiomes in each group at 3 d after 13 Gy TAI. (d) Linear discriminant analysis (LDA) effect size (LEfSe) of key genera that contribute to differences in the structure of mucosal microbiota between the control, IR + vehicle and IR + I3A groups. Only taxa meeting an LDA threshold > 3 were shown. (e) Hierarchical clustering heatmap of gut microbiota in the feces of all 3 groups. Red represents increased expression, while blue represents decreased expression. Relative abundances of *Lactobacillus* (f), *Bifidobacterium* (g), *Alloprevotella* (h), *Lachnospiraceae_NK4A136* (i), *Desulfovibrio* (j) and *Escherichia-shigella* (k) in each group. Data are presented as the mean ± S.D., *p < .05; **p < .01; ***p < .001; *n* = 6/group.

To investigate the effects of IR and microbiota on I3A, we tested I3A fecal extracts of specific pathogen free (SPF) and germ-free (GF) mice using high performance liquid chromatography (HPLC). Notably, the fecal I3A content was increased after 13 Gy TAI in SPF mice, while the increment was completely reversed in GF mice, suggesting that IR-induced I3A production depends on gut microbiota (Figure 4a).

Given that gut microbiota configurations relate to RIE progression, we addressed the effects of I3A on the alterations in intestinal bacterial structure in TAI-exposed male mice. Fecal pellets of vehicle or I3A-treated mice were collected on day 3 after TAI and subjected to 16S rRNA sequencing. Based on alpha diversity analysis and principal coordinate analysis (PCoA), gut microbiota structures were substantially altered after TAI and I3A treatment (Figure 4b,c). There were significant differences in taxon abundance between the IR + vehicle and IR + I3A treatment cohorts at the genus level (Figure 4d, e). The relative abundance of the genera *Lactobacillus*, *Bifidobacterium*, *Alloprevotella* and *Lachnospiraceae_NK4A136*, which are widely known for their benefits for human health, were enriched in irradiated mice treated with I3A (Figure 4f–i). Conversely, I3A treatment reduced the relative abundance of pathogenic bacteria, such as *Desulfovibrio* and *Escherichia-Shigella* (Figure 4j, k). Taken together, these data confirmed that I3A enhanced the relative abundance of probiotics while reducing the abundance of pathogenic bacteria after 13 Gy TAI exposure.

### Beneficial effects of I3A are dependent on AhR/IL-10

**Figure 5.**
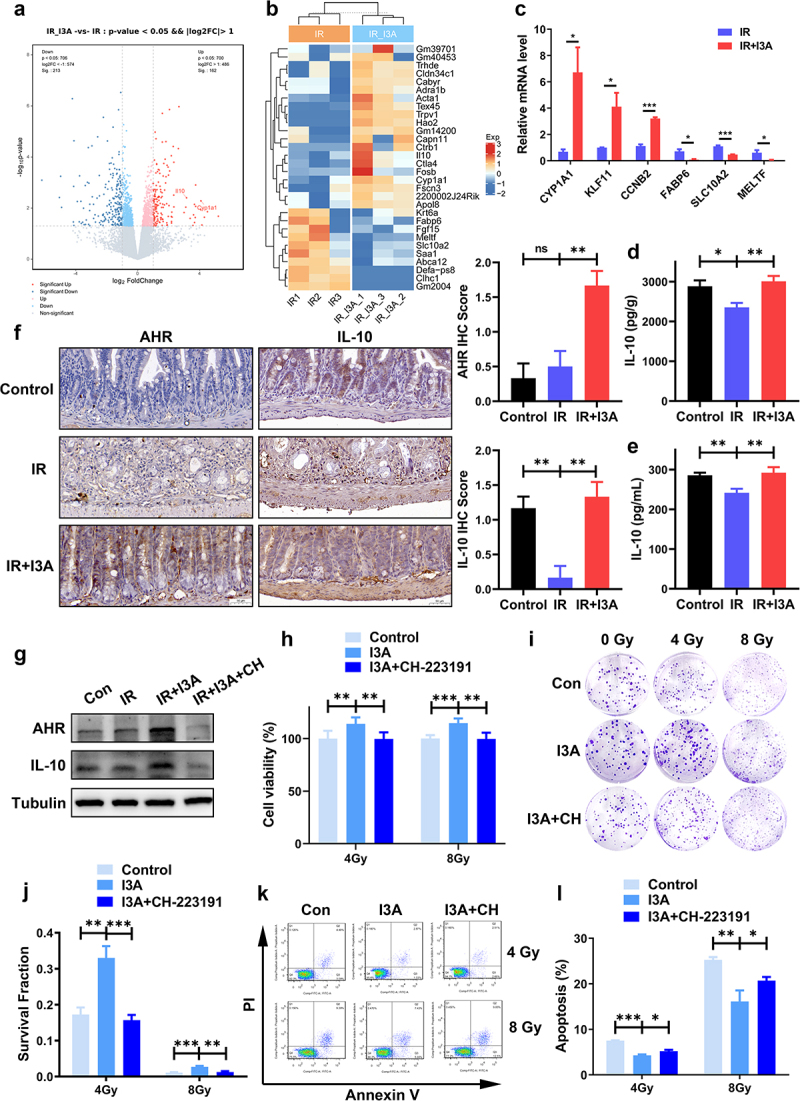
The beneficial effect of I3A involves the AhR/IL-10 axis. (a) Volcano plot showing the DEGs between vehicle-treated *vs*. I3A-treated samples at 6 h post-TAI (*n* = 3/group). Genes with a fold change ≥ 2 were marked with red dots, and those with a fold change ≤ 0.5 were marked with blue dots. (b) Gene cluster map of the DEGs. (c) Quantitative RT-PCR validation of the DEGs. *p < .05; **p < .01; *n* = 3/group. (d-e) The concentration of IL-10 in intestinal tissues and serum. (f) Representative IHC images showing AhR and IL-10 expression in the small intestines of vehicle- and I3A-treated mice (scale bar = 50 μm); IHC scores were determined as described in materials and methods. (g) Verification of inhibition of AhR expression by its inhibitor CH-223191. Proteins of HIEC-6 cells pretreated with 100 μM I3A or with 10 μM CH-223191 were extracted, and Western blotting was performed to detect the protein levels of AhR and IL-10. Tubulin was used as a loading control. CH: CH-223191. (h) CCK-8 assay of HIEC-6 cells pretreated with vehicle, 100 μM I3A or 100 μM I3A +10 μM CH-223191 following 4 or 8 Gy radiation. (i-j) Colony formation of HIEC-6 cells treated with vehicle, 100 μM I3A or 100 μM I3A +10 μM CH-223191 subjected to 0, 2, 4, 6, and 8 Gy X-ray radiation. (k-l) Cell apoptosis was measured using Annexin V/PI staining in HIEC-6 cells pretreated with 100 μM I3A, and 10 μM CH-223191 following 4 or 8 Gy IR. Data are presented as the mean ± S.D. of three independent experiments. *p < .05; **p < .01; ***p < .001.

To further discern the underlying mechanisms that contribute to I3A-mediated radioprotection, we profiled the small intestine transcriptomes of the different treatment groups and identified 375 differentially expressed genes (DEGs) between the IR+vehicle- and IR+I3A-treated mice, of which 162 genes were upregulated and 213 were downregulated (Figure 5a,b). To validate the results of transcriptome sequencing, a literature review of the top 30 upregulated and downregulated genes was conducted to screen for genes related to radiation-induced injury. Ultimately, the expression patterns of 6 representative genes (three upregulated genes – cytochrome P450 1A1 (CYP1A1), Krüppel-Like Factor 11 (KLF11) and cyclin B2 (CCNB2), and three downregulated genes – fatty acid binding protein 6 (FABP6), solute carrier family 10, member 2 (SLC10A2) and melanotransferrin (MELTF)) were validated by qRT-PCR, and all results were consistent with the sequencing data (Figure 5c).

Administration of I3A significantly increased the mRNA level of CYP1A1, which is the main downstream target of the transcription factor AhR. Its mRNA level can directly reflect whether AhR is activated. Moreover, I3A induced interleukin-10 (IL-10) gene and protein expression (Figure 5b,d,e). Correspondingly, IHC staining further showed that treatment with I3A significantly improved the expression levels of AhR and IL-10 *in vivo* (Figure 5f). Western blotting further verified this result in HIEC-6 cells (Figure 5g).

To further confirm the role of AhR in regulating the radioprotective effects of I3A, we used CH-223191, a potent and specific antagonist of AhR, to suppress nuclear translocation and DNA binding of AhR. Western blot analysis showed that CH-223191 effectively inhibited AhR/IL-10 expression in HIEC-6 cells (Figure 5g). CCK-8 and clonogenic survival assays showed that CH-223191 almost completely abolished I3A-mediated radioprotection in HIEC-6 cells (Figure 5h–j). Furthermore, CH-223191 partially blocked the cytoprotective effects of I3A on IR-induced apoptosis, as shown by Annexin V/PI staining (Figure 5k, l). These results indicate that the radioprotective effects of I3A against RIE depends on AhR/IL-10 signaling.

### I3A regulates ISCs proliferation and differentiation through AhR/IL-10/Wnt3

**Figure 6.**
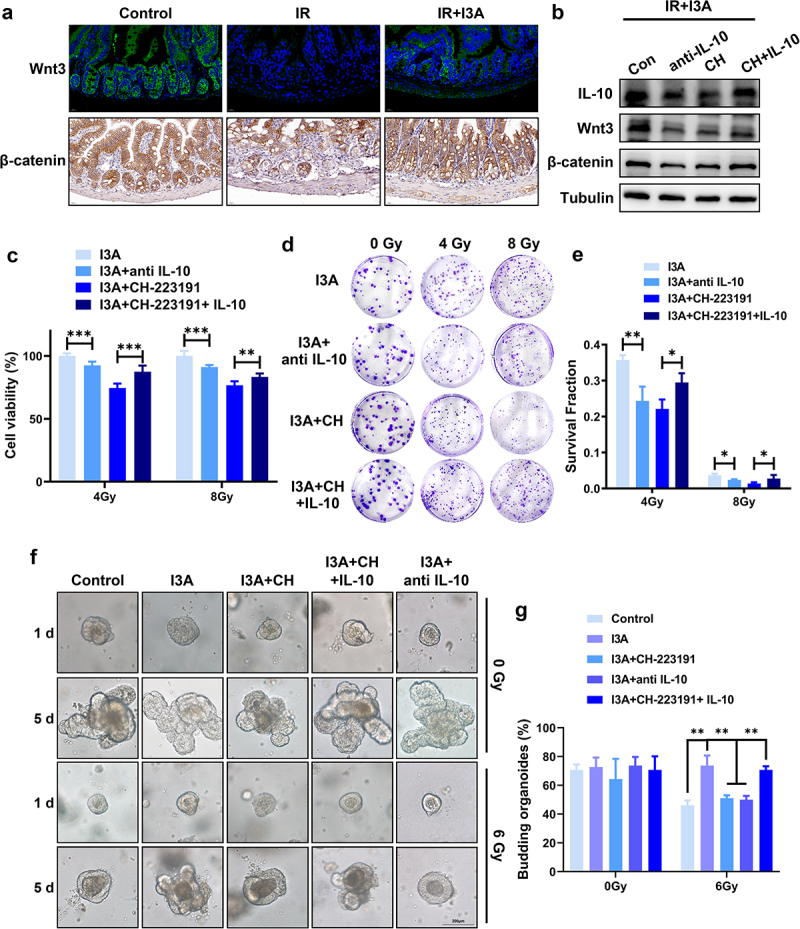
I3A activates the Wnt3/β-catenin signaling pathway. (a) Representative IF and IHC images showing Wnt3 and β-catenin expression in the small intestines of vehicle- and I3A-treated mice at 3 d post-TAI (scale bar = 20 μm). (b) Western blot results of IL-10, Wnt3 and β-catenin in HIEC-6 cells pretreated with I3A (100 μM) or CH-223191 (10 μM) with/without IL-10 (5 ng/ml) or anti-IL-10 (0.1 μg/ml) following 4 Gy IR. (c) CCK-8 assay of HIEC-6 cells pretreated with 100 μM I3A, 100 μM I3A +0.1 μg/ml anti-IL-10, 100 μM I3A +10 μM CH-223191 or 100 μM I3A +10 μM CH-223191 + 5 ng/ml IL-10 following 4 or 8 Gy radiation. (d-e) Colony formation results of HIEC-6 cells pretreated with 100 μM I3A, 100 μM I3A +0.1 μg/ml anti-IL-10, 100 μM I3A +10 μM CH-223191 or 100 μM I3A +10 μM CH-223191 + 5 ng/ml IL-10 for 1 h and then subjected to 0, 2, 4, 6, and 8 Gy X-ray radiation. (f) Representative phase contrast images of intestinal organoids in different groups at 1 or 5 d post-IR (scale bar = 200 μm). Mouse intestinal crypt organoids treated with vehicle, 100 μM I3A and/or 10 μM CH-223191, 0.1 μg/ml anti-IL-10 and 5 ng/ml IL-10 for 1 h and then subjected to 0 or 6 Gy X-ray radiation. (g) The budding organoids percentage of total organoids per well at 5 d post-IR. Data are presented as the mean ± S.D. of three independent experiments. **p < .05; **p < .01; ***p < .001.

Intestinal epithelial self-renewal, homeostasis, and repair are dependent upon Wnt-β-catenin signaling. Activation of Wnt-β-catenin signaling translocates β-catenin to the nucleus to switch on a series of genes that support ISCs proliferation and differentiation.^31^ We analyzed the effect of I3A on crypt epithelial Wnt-β-catenin activation. Mice receiving I3A displayed a significant increase in Wnt3- and β-catenin-positive cells compared with irradiated mice (Figure 6a). Western blotting further showed that I3A-stimulated Wnt3-β-catenin activation was dependent on IL-10 in irradiated HIEC-6 cells, as evidenced by decreased expression of Wnt3 and β-catenin in response to anti-IL-10 stimulation and increased expression of Wnt3 and β-catenin in response to IL-10 supplementation even in the presence of CH-223191 (Figure 6b). CCK-8 and clonogenic survival assays showed that IL-10 supplementation could counteract the inhibitory effects of CH-223191 on I3A-mediated radioprotection in HIEC-6 cells (Figure 6c-e).

To further determine the influence of I3A on radiation-induced intestinal injury and related mechanisms, these results were confirmed at the mouse intestinal organoid level (Figure 6f, g). In addition, we confirmed that the regulation of intestinal proliferation and differentiation by I3A at the organoid level depends on AhR/IL10/Wnt3 using IF (Figure S2). Notably, the activation of Wnt/β-catenin signaling, illustrated in Figure 6a, triggered a cascade of molecular events associated with increased stem cell activity and proliferation. This enhanced the proliferative state, governed by Wnt/β-catenin signaling, and was manifested as the budding phenomenon shown in Figure 6f. Overall, I3A treatment promoted the damage repair of intestinal epithelial cells in an AhR/IL-10/Wnt3/β-catenin pathway-dependent manner.

### I3A does not protect malignant tissues against radiation

**Figure 7.**
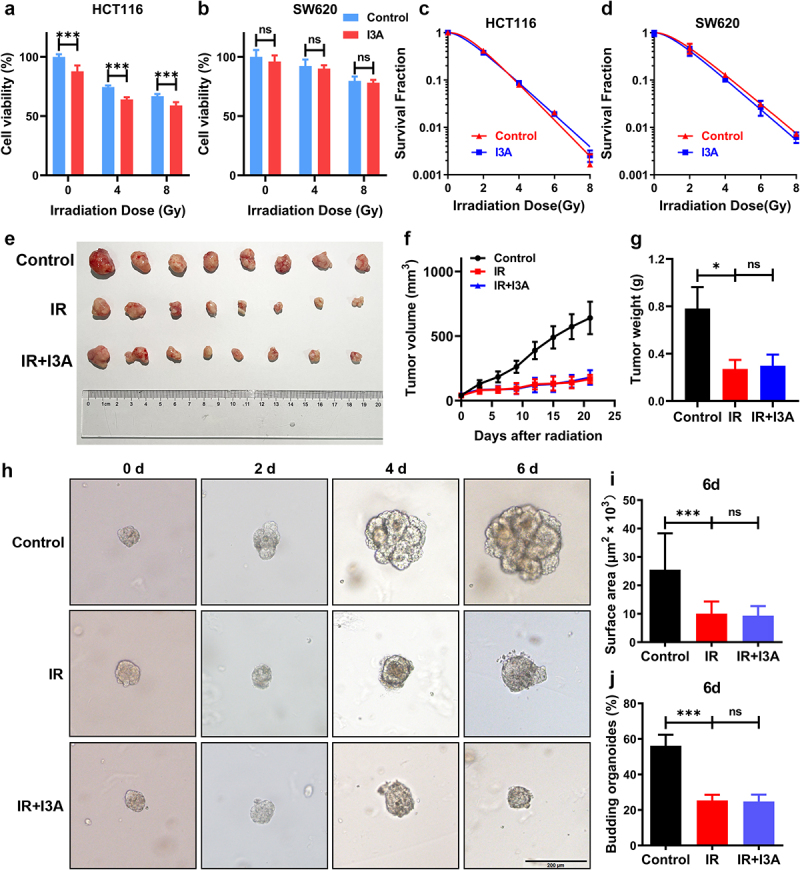
I3A does not protect malignant tissues against radiation. (a, b) Effects of I3A on cell viability of HCT116 and SW620 cells post IR. HCT116 and SW620 cells were pretreated with 100 μM I3A 1 h before 4 or 8 Gy IR, and cell viability was measured after 24 h using the CCK-8 assay. (c, d) Clonogenic survival assay of HCT116 and SW620 cells pretreated with 100 μM I3A or vehicle 1 h following 0, 2, 4, 6, or 8 Gy radiation. (e) After 21 days, the mice were sacrificed, and the images of the tumors in each group were captured (*n* = 8 per group). (f) Tumor volume was recorded over time throughout the experiment. (g) Tumor weight in individual mice was detected. (h) Representative phase contrast images of human colorectal cancer organoids cultured with or without I3A at 0, 2, 4 or 6 d post-IR (scale bar = 200 μm). (i) Surface area per organoid at 6 d post-IR. At least 50 colorectal cancer organoids were counted in each group. (j) The budding organoids percentage of total organoids per well on day 6. At least 50 colorectal cancer organoids were counted in each group. Data are presented as the mean ± S.D. of three independent experiments. *p < .05; ***p < .001.

I3A treatment at a concentration of 100 μM showed a cytotoxic effect on cell proliferation in HCT116 cells, and colony formation assays did not reveal an increase in the survival and clonogenic potential of irradiated HCT116 cells upon I3A treatment (Figure 7a,c). I3A treatment also showed no radioprotection in SW620 cells (Figure 7b, d).

A colorectal cancer xenograft mouse model was established to further evaluate the protection effect of I3A against radiation *in vivo*. Subcutaneous tumors were developed by injecting HCT116 colon cancer cells into the mouse flanks. Mice with palpable subcutaneous tumors were exposed to IR (10 Gy), followed by treatment with or without I3A. IR significantly inhibited tumor growth compared to the control; however, there was no significant difference in the growth of ectopic tumors between the IR and the IR+I3A groups (Figure 7e–g).

Moreover, at the human colorectal cancer organoid level, I3A treatment (100 μM) 1 h before radiation exposure (6 Gy) did not rescue organoids from radiation toxicity. Within 4–6 days post-irradiation, the budding crypts sharply reduced in both I3A-treated and vehicle-treated organoids (Figure 7h, i). Additionally, I3A did not affect the surface area of malignant organoids, suggesting that I3A does not have a radioprotective effect in malignant tissues (Figure 7j).

These results clearly suggest that I3A has no protective effect on tumors during radiotherapy; therefore, I3A is a promising medical countermeasure against RIE for clinical use during pelvic radiotherapy.

## Discussion

The human gut microbiota is a complex microbial ecosystem composed of approximately 1,000 different species of microorganisms. Microbiota has been emphasized as an important contributor to the complex nature of diseases in recent years. Microbiota can influence gastrointestinal barrier function and host immune response through metabolic activities such as SCFAs from carbohydrate metabolism and tryptophan metabolites from amino acid metabolism after establishing themselves in the intestine.^32,33^ Various metabolites such as SCFAs, indole derivatives, and other gut microbiota metabolites have been reported to prevent or alleviate RIE.^34^ Gut microbiota metabolite IPA protects against radiation-associated hematopoietic and gastrointestinal syndrome.^18^ However, it is still uncertain which Trp metabolites show the best radioprotective effect in the context of RIE. Recently, three-dimensional (3D) *ex vivo* organoid technology has been developed to mimic physical and biochemical features of *in vivo* tissues. The *ex vivo* intestinal crypt organoids have been used as a mean to monitor a number of biological responses to pathophysiological insults or treatments and used as model systems for screening drugs.^35^ In this study, we screened the radioprotective efficacy of Trp metabolites at the intestinal organoid level and found that I3A had a significant therapeutic effect. Furthermore, I3A treatment significantly prolonged survival time in mice after TAI and reversed radiation-induced intestinal damage.

Intestinal epithelial cells regenerate rapidly, making the small intestine vulnerable to irradiation.^36^ Epithelial injury induced by IR results primarily from the elimination of ISCs at the bottom of the crypt.^37^ In addition to stem cells in the crypt and their transit amplifying daughter cells, the intestinal epithelium contains terminally differentiated functional cells, such as paneth, goblet, enteroendocrine, enterocytes, and tuft cells.^38^ By secreting antimicrobial molecules, paneth cells protect the epithelium and maintain it by providing an epithelial niche for ISCs.^39^ Several studies have suggested that Lgr5^+^ ISCs are important in gut regeneration following radiation exposure.^25^ According to our study, I3A is closely related to the Lgr5^+^ ISCs population in the intestine in mediating radioprotective effects, as pretreatment with I3A reduced the loss of Lgr5^+^ ISCs caused by radiation injury. Correspondingly, it was further observed that I3A increased the number of Ki67^+^ progenitors, paneth cells and goblet cells. Based on these findings, I3A therapy preserves the normal architecture of the small intestinal crypt-villus structure.

Epithelial cells cover the mucosa of the intestine to protect it from the external environment and provide a barrier to the internal environment. Radiation damages the intestinal epithelium and leads to oxidative stress, which disrupts tight junctions, adherens junctions, and the actin cytoskeleton of the intestinal epithelium.^40^ This is consistent with our results; after IR exposure, mice exhibited decreased levels of tight junction proteins, including ZO-1, Occludin, Claudin-3, and E-cadherin. The indole metabolite I3A, produced by the microbiome from Trp catabolism, has positive effects on the immune system and barrier function.^41^ I3A supplementation may protect against immune checkpoint inhibitors-induced colitis by maintaining epithelial barrier integrity and dampening the inflammatory response.^41, 42^ In this study, we showed that I3A promotes intestinal tight junction protein expression and maintains intestinal permeability.

Gut microbiota composition is an important factor contributing to the regulation of intestinal mucus barrier function. Dysbiosis of intestinal flora is linked to the development of IR-induced injuries. Our previous research found that IR exposure caused intestinal microbiota dysbiosis, which reduced microbiota diversity and abundance, increased the population of pathogenic bacteria, and decreased the population of probiotics.^43^ In this study, we found that I3A could increase the relative abundance of radioprotective *Bifidobacterium* and *Lactobacillus* and decrease the abundance of pathogenic bacteria. *Lactobacillus* species colonize most sites in the gastrointestinal tract and are well-known probiotics for their multiple benefits, including regeneration of the intestinal epithelium through the production of lactic acid.^44^ Furthermore, *Bifidobacterium* and *Lactobacillus* have been reported to have potential benefits in improving symptoms associated with RIE and benefiting intestinal homeostasis.^44–46^ I3A could decrease the abundance of pathogenic bacteria, such as *Desulfovibrio* and *Escherichia-Shigella*. *Desulfovibrios* produce sulfide, which is toxic to colonic epithelial cells and have previously been linked to ulcerative colitis.^47^ *Escherichia-Shigella* can invade colonic epithelium and provoke strong inflammatory responses, leading to the destruction of colonic epithelium, which then results in bacillary dysentery.^48^ In addition, this study found that the gut microbiota can drive the expression of I3A, and in turn, supplementation with I3A can regulate the balance of the gut microbiota, indicating that there is a complex bidirectional interaction between gut microbiota metabolites and gut microbes.

In this study, we showed that the gut microbiota metabolite I3A benefits ISCs self-renewal and intestinal regeneration. However, the mechanism by which I3A regulates ISCs proliferation and differentiation remains to be fully elucidated. Many types of cells contain AhR, which is a ligand-activated transcription factor containing basic helix-loop-helix domains, as the receptor for I3A.^49^ Upon translocation into the nucleus, AhR induces the expression of downstream target genes, such as CYP1A1 and CYP1B1. As a powerful transcription factor, AhR mainly regulates the transcription and secretion of various cytokines, including immune regulatory factors such as IL-6, IL-10, and IL-22, to maintain intestinal tissue homeostasis.^22,50^ In this study, we demonstrated that I3A increased the secretion of IL-10 to accelerate ISCs-mediated epithelial regeneration. The induction of IL-10 by I3A was attributed to the activation of AhR, which was further confirmed by treatment with an AhR inhibitor. This explanation was further confirmed by the addition of anti-IL-10, which resulted in fewer budding organoids.

Intestinal epithelial homeostasis and repair are primarily maintained by ISCs self-renewal and proliferation, which is the result of Wnt-β-catenin signaling.^51^ Wnt ligands are critical for activating Wnt-β-catenin signaling and rescuing these stem cells following radiation injury.^52^ We found that I3A stimulated the Wnt3/β-catenin signaling pathway, which could be inhibited by the addition of anti-IL-10. Furthermore, I3A activates IL-10-Wnt-β-catenin signaling in irradiated crypts to induce crypt stem cell proliferation and regeneration. Moreover, the IL-10-Wnt-β-catenin signaling in intestinal antigen-presenting cells has been reported to protect mice from colitis-associated colon cancer.^53^

Interestingly, I3A does not appear to affect colon cancer cells after radiation exposure, even though it can protect ISCs by reducing radiation-induced apoptosis. For patients with gastrointestinal malignancies who undergo abdominal irradiation, systemic I3A may be beneficial during radiotherapy. Indole derivatives, including I3A, exhibit diverse biological activities and have been reported to inhibit tumor development and possess chemopreventive properties against cancer. ,^54–56^ For instance, indole-3-lactic acid has been reported to enhance CD8^+^ T cell-mediated immune responses against colorectal tumors, while IPA has demonstrated protection against radiation toxicity in mice without precipitating tumor growth.^18,57^ However, the specific reasons why I3A did not protect colon cancer cells in culture may be attributed to complex interactions with tumor cells. This could involve specific cellular pathways or molecular targets that are either inadequately expressed or unresponsive in these particular cell lines. Additionally, genetic variations or phenotypic differences unique to these colon cancer cells may influence their susceptibility to I3A treatment. Further investigation into the molecular mechanisms underlying the differential response of normal and tumor cells to I3A treatment is essential. Understanding these mechanisms could facilitate the development of more effective strategies to mitigate radiation-induced damage to normal tissues while simultaneously enhancing the therapeutic efficacy of radiotherapy against tumors.

This study has some limitations. First, it focused exclusively on some common indole derivatives, omitting an analysis of all tryptophan metabolites influenced by the gut microbiota. Given the well-documented bioactivities of indole and other derivatives, future studies should explore their potential effects on radiation-induced intestinal injury. Another limitation lies in the potential challenges associated with reproducing our results in true cancer models. The tumor microbiome, also known as the intratumoral microbiome, is a crucial component of the tumor microenvironment, considerably impacting tumor occurrence, development, and treatment.^58^ As such, it can influence cancer transformation by affecting the host genome, inducing a pro-inflammatory microenvironment, and modulating metabolites, among other factors. It also plays a role in shaping the immune environment and promoting tumor growth.^59^ The pro-inflammatory microenvironment and the diverse microbial compositions observed in tumor models may affect the reproducibility of our findings, influencing the actions of I3A and potentially leading to the preferential metabolism of less bioactive metabolites. Consequently, a cautious interpretation of our results within the broader context of cancer models is crucial.

**Figure 8.**
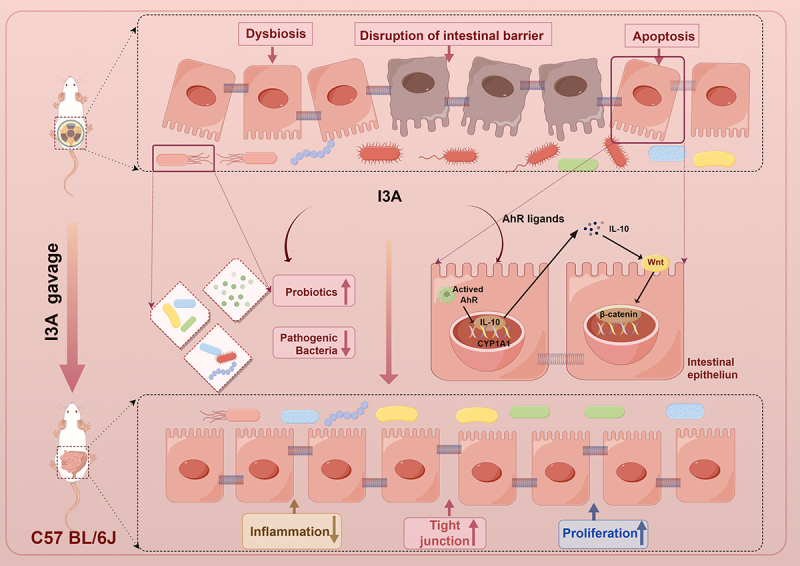
Gut microbiota – derived I3A protects the intestine against radiation injury. Exposure to ionizing radiation results in an imbalance of the intestinal flora, impairment of the integrity of the intestinal barrier, and induction of inflammation. However, the administration of I3A has been found to enhance the relative abundance of probiotics while reducing the abundance of pathogenic bacteria. Consequently, this intervention partially restored the diversity of the gut microbiota and its dysbiosis and facilitated the maintenance of a tightly interconnected intestinal barrier. Simultaneously, I3A stimulated intestinal epithelial cells to secrete IL-10 via AhR activation and then IL-10 activated Wnt3/β-catenin signaling to accelerate intestinal epithelial proliferation regeneration and thus maintain the epithelial barrier. This figure was drawn by Figdraw (www.figdraw.com).

In summary, we demonstrated that microbiota-derived I3A can protect against RIE and microbiota dysbiosis by improving mucosal barrier functions. In addition, I3A induces AhR/IL-10/Wnt signaling activation and contributes to the impacts of proliferation and differentiation of ISCs (Figure 8). I3A did not have any radioprotective effect on abdominal tumors and therefore could improve the therapeutic efficacy of abdominal radiotherapy. Our results highlight that I3A might be used as an effective radioprotective agent in individuals with RIE.

## Materials and methods

### Chemical sources and preparation

I3A, IAA, IPA, ILA, skatole and tryptamine were purchased from Sigma-Aldrich (St Louis, MO, USA). For the *in vitro* experiments, I3A was dissolved in dimethylsulfoxide (DMSO, Solon, OH, USA) at a stock concentration of 100 mM. For the *in vivo* experiments, I3A was dissolved in DMSO at a stock concentration of 0.5 mg/mL. The AhR inhibitor 1-methyl-N-[2-methyl-4-[2-(2-methylphenyl)diazenyl]phenyl-1 H-pyrazole-5-carboxamide (CH-223191) was purchased from AbMole (Shanghai, China), dissolved in DMSO, and prepared into a 10 mM stock solution. Recombinant human IL-10 (5 ng/mL; HZ-1145) and anti-IL-10 neutralizing antibody (0.1 μg/mL; 69018-1-1 g) were purchased from Proteintech (Chicago, IL, USA).

### Mice

Male C57BL/6J mice (6–8 weeks old) and BALB/c nude mice (3–5 weeks old) were purchased from Shanghai SLAC Laboratory Animal Co. Ltd. (Shanghai, China). Mice were housed in the SPF level animal facility at the Animal Centre of Soochow University. Mice were kept under standard conditions (ambient temperature, 22 ± 2°C; air humidity, 40–70%; and a 12/12-h light/dark cycle) with continuous access to a standard diet and water. All animal experiments were conducted following protocols approved by the Animal Ethics Committee of Soochow University (Approval no.: SUDA202112A1098, SUDA202308A0634).

The GF male C57BL/6J mice (6–8 weeks) were bred and maintained in special plastic isolators (GemPharmatech, Nanjing, China). All mice were housed under a strict 12-hour light cycle (lights on at 08:00). Animals were supplied with a 50-kGy irradiated sterile pelleted normal chow diet (Xietong Shengwu, Nanjing, China) and autoclaved tap water *ad libitum*. Bedding was replaced in all experiments every 7 d. All GF mice were routinely screened for bacterial, viral, and fungal contamination (Approval no.: GPTAP20230511–1).

### Intestinal organoid isolation and treatments

Intestinal organoid isolation and treatments was performed as described in our previous study.^60^ The resulting organoids were treated with complete medium with or without 100 μM I3A, IPA, ILA, IAA, tryptamine, or Skatole 1 h pre-IR and then exposed to 6 Gy X-rays at a dose rate of 1.1 Gy/min or sham-irradiated.

The surface areas of the organoids were measured microscopically by capturing multiple random, non-overlapping images of the organoids in a well, using an inverted microscope from Olympus Corp., Tokyo, Japan. Organoid perimeters for area measurements were determined both manually and through automated analysis using the Analyze Particle function of ImageJ software. The sizes of the largest and smallest organoids in the reference well were measured manually; their corresponding areas served as the reference values for establishing the minimum and maximum particle sizes. Organoids that were in contact with the edges of the images were excluded from counting. Following cultivation, the numbers of budding and disrupted organoids per well were manually determined using light microscopy to assess growth efficiency. Budding organoids represent the outgrowth or budding process occurring during the proliferation and differentiation of ISCs within the organoids. In contrast, disrupted organoids indicate organoids that have disintegrated in response to radiation, with the presence of debris in their vicinity. At least 50 organoids per group were counted. For IF, organoids were fixed with 4% paraformaldehyde (PFA) for 1 h and fixed to prepare frozen sections, then incubated with primary antibodies.

### Cell culture

HIEC-6, HCT116 and SW620 cells were purchased from the American Type Culture Collection (ATCC, Manassas, VA, USA). The cells were cultured with 10% fetal bovine serum (Biological Industries, Cromwell, CT, USA), and 1% (v/v) penicillin‒streptomycin (Beyotime, Shanghai, China) and grown at 37°C in a humidified 5% CO_2_ atmosphere.

### Cell viability assay

The cell viability was evaluated using a cell counting kit-8 (CCK-8; Beyotime, Shanghai, China) as per the manufacturer’s instructions. The cells (2000–10000 cells/well) were first seeded into 96-well plates and incubated for 24 h. The cells were then subjected to varying treatments. CCK-8 solution (10 μL) was then added to each well, followed by incubation for 2 h at 37°C. The optical density (OD) of the cells was finally measured at 450 nm using a microplate reader (BIO-TEK, Winooski, VT, USA).

### LDH leakage assay

Cell death was evaluated using the Lactate Dehydrogenase (LDH) Cytotoxicity Assay Kit (Beyotime, Shanghai, China). The HIEC-6 cells were grown in 96-well plates for 24 h and subsequently subjected to various treatments. The level of LDH in the cell culture supernatant was measured according to the manufacturer’s instruction.

### Colony formation assay

Cells were seeded in 6-well plates in triplicate at densities of 200–2000 cells/well depending on the radiation dose. The cells were cultured overnight, treated with or without 100 μΜ I3A for 1 h, and then subjected to 0, 2, 4, 6, and 8 Gy X-ray radiation. The medium containing drugs was then immediately replaced with fresh medium, and the cells were subsequently cultured at 37°C for 1–2 weeks to form colonies. The cell colonies were stained with crystal violet and viewed under a microscope. Viable colonies consisting of at least 50 cells were counted.

### Cell apoptosis assay

Annexin V/PI double-staining analysis was used to detect cell apoptosis. The cells were seeded in triplicate in 6-well plates, cultured overnight and pretreated with 0 or 100 μM I3A for 1 h before 0, 4 or 8 Gy irradiation. After 48 h, the cells were stained with FITC-conjugated Annexin V and PI (KeyGen, Nanjing, China). Apoptosis analysis was performed using a FACSVerse flow cytometer (BD Biosciences, San Diego, CA, USA).

### RIE mouse model establishment

All mice were subjected to 13 Gy TAI using the X-RAD 320iX Biological Irradiator (Precision X-ray, North Branford, CT, USA) at a dose rate of 1.0 Gy/min. The whole abdomen was irradiated and the other parts of the mouse were shielded using lead. Control mice were sham-irradiated.

Mice were randomly divided into two groups (*n* = 20/group), IR and IR+I3A, for survival experiments. I3A (200 mg/kg) was administered by oral gavage into SPF C57BL/6J mice in three doses: 1 d before radiation, 1 h before radiation, and 1 d after radiation (Figure 2a). Mice in the IR group received an equal volume of DMSO by oral gavage at the same frequency as the IR+I3A-treated mice. The mice were randomly divided into 3 groups (*n* = 6/group), Control, IR, and IR+I3A, for subsequent experiments. A clinical score was determined using a cumulative scoring system (Supplementary Table S1) based on weight loss, temperature change, physical appearance, posture, mobility, food consumption, and hydration.

### Histopathology

The mouse intestines were harvested at 6 h or 3 d after TAI and fixed in 4% PFA overnight at room temperature and then embedded in paraffin. Tissues were sectioned at 5 μm thickness for hematoxylin-eosin (H&E) staining and periodic acid-Schiff (PAS) staining using standard protocols. H&E-stained sections were viewed under an optical microscope (Olympus Corp., Tokyo, Japan), followed by analysis of the villus height and mucosal thickness using ImageJ software; values were initially recorded in pixels and subsequently converted to micrometer units using the conversion factor 1.46. The histological score was determined using a cumulative scoring system (Supplementary Table S2) and performed in a double-blinded fashion by a licensed pathologist.

IHC and IF were performed as described by Li M et al.^60^ The primary antibodies used for IHC staining included anti-Lgr5 (1:200; 251487; Abbiotec, San Diego, CA, USA), anti-Ki67 (1:400; CST12202; Cell Signaling Technology, Beverly, MA, USA), anti-AhR (1:200; 67785; Proteintech, Chicago, IL, USA), anti-IL-10 (1:100; 69018; Proteintech) and anti-β-catenin (1:400; CST8480; Cell Signaling Technology) antibodies. The primary antibodies used for IF staining included anti-Lysozyme (1:200; NBP2–6118; Novus Biological, Littleton, CO, USA), anti-Wnt3 (1:50; ab50341; Abcam, Cambridge, MA, USA), anti-E-cadherin (1:200; CST14472; Cell Signaling Technology), anti-ZO-1 (1:50; 61–7300; Invitrogen, Carlsbad, CA, USA), anti-Occludin (1:50; 40–4700; Invitrogen) and anti-Claudin 3 (1:100; 34–1700; Invitrogen) antibodies. For the quantification of IF staining intensity, images were processed and analyzed with ImageJ software. Matched images, captured under the same exposure, were subjected to identical processing and analysis. The two-dimensional image intensities of fluorescence (in pixels) of *n* = 6 randomly selected fields in micrographs from each condition were measured using ImageJ software and graphically represented in GraphPad Prism. The IHC Profiler plugin in ImageJ software was employed for analysis, combining the average grayscale value (staining intensity) and the percentage of positive cells (staining area) as IHC measurement indicators.^61^ The results were then categorized into four ratings – High positive (3+), Positive (2+), Low positive (1+), and Negative (0).

### TUNEL assay

The intestine sections at 6 h post-TAI were stained using an *in situ* Cell Death Detection Kit (Roche Diagnostics, Mannheim, Germany) according to the manufacturer’s instructions.

### In vivo intestinal permeability assay

Mice were randomly divided into 3 groups (*n* = 3/group), Control, IR, and IR+I3A, and treated as described earlier. On day 1 after radiation, mice were fasted but allowed access to water for 4 h. FITC-dextran (50 mg/100 g body weight; 4 kD 46,944; Sigma) was given orally. At 2 h after gavage, blood was collected, and the fluorescence of serum samples was measured using a microplate reader (excitation, 490 nm; emission, 520 nm). The fluorescence intensity of each sample was normalized to the average value of the non-radiation group.

### Quantification of I3A

Quantification of I3A in supernatants of fecal samples was performed by HPLC. We weighed 50 mg of each sample and placed them into 2 mL stoppered tubes and added 0.5 mL of 2 mol/L trifluoroacetic acid. The samples were then hydrolyzed for 8 h in a water bath at 100°C; then, the hydrolyzates were neutralized with 4 mol/L sodium hydroxide solution to neutral, and diluted to 1 mL. We centrifuged the clear solution and mixed 200 μL of the solution with 100 μL 0.5 mol/L PMP-methanol solution and 100 μL 0.3 mol/L NaOH solution and incubated in a water bath at 70°C for 30 min. Subsequently, 100 μL 0.3 mol/L HCl was added for neutralization and 1 mL chloroform was added for extraction. Following filtration through a 0.45 μm membrane, an equal volume of samples was loaded onto an HPLC (Thermo Fisher U3000 liquid chromatograph) chromatographic column: C18 AQ (250 × 4.6 mm; 5 μL); sulfuric acid (0.01 M) was used as the mobile phase, and the levels of I3A were determined by the external standard calibration method.

### Enzyme-linked immunosorbent assay (ELISA)

Intestinal tissues were lysed with phosphate buffer saline (PBS) and homogenized to extract the total proteins in a homogenization device (Leica, Wetzlar, Germany) under precooled conditions. The expression levels of IL-10 in intestinal tissue supernatants or sera were quantified with commercially available ELISA kits (ZCIBIO Technology Co. Ltd, Shanghai, China) as per the manufacturer’s directions.

### High-throughput 16S rRNA gene amplicon sequencing and analysis

The fecal samples of each mouse were freshly collected at 3 d after TAI and then immediately placed into sterile plastic tubes on ice and frozen at − 80°C. 16S rRNA sequencing was performed as described in our previous study.^62^ The raw data were uploaded to the NCBI Sequence Read Archive (SRA) database under the BioProject accession number PRJNA1020984. Species annotation analysis was conducted using the Mothur method and SSUrRNA Database. Alpha and beta diversity analyses were determined for each library using QIIME software (Version 1.9.1). In the alpha diversity analysis, Wilcoxon indices were used to evaluate the richness and evenness of the gut microbiota and the complexity of its structure. The PCoA is commonly used in beta diversity analysis to determine the repeatability of samples within a group and similarities and differences between different groups. Linear discriminant analysis (LDA) effect size (LEfSe) software was used to perform LEfSe analysis.

### Transcriptome sequencing and bioinformatics analysis

Intestinal tissues were isolated from the I3A- or vehicle-treated mice (*n* = 3/group) at 6 h after 13 Gy TAI, and total RNA was extracted using the RNA Isolation Kit (Ambion, Austin, TX, USA) according to the manufacturer’s protocol. RNA integrity was assessed using the Agilent 2100 Bioanalyzer (Agilent Technologies, Santa Clara, CA, USA), and samples with an RNA integrity number (RIN) greater than 7 were used for subsequent analysis. The libraries were constructed using the TruSeq Stranded mRNA LT Sample Prep Kit (Illumina, San Diego, CA, USA) according to the manufacturer’s instructions, and transcriptome sequencing was performed using the Illumina sequencing platform by Shanghai OE Biotech. Co. Ltd. (Shanghai, China). The raw data were uploaded to the NCBI Sequence Read Archive (SRA) with accession number PRJNA1020982.

### Quantitative real-time PCR (qRT‒PCR) analysis

qRT-PCR was performed as described in our previous study.^60^ The expression levels of CYP1A1, KLF11, CCNB2, FABP6, SLC10A2, and MELFT mRNAs were measured by real-time PCR in triplicate using the 7500 Real-Time PCR system (Applied Biosystems, Foster City, CA, USA). The expression levels of the target genes were normalized to β-actin. The primers were synthesized by Sangon Biotech (Shanghai, China) and are listed in Supplementary Table S3.

### Western blot analysis

Protein levels of HIEC-6 cells were analyzed using the Western blot assay according to the published methods.^62^ The primary antibodies used were anti- AhR (1:2000; 67785-1-1 g; Proteintech, Chicago, IL, USA), anti-IL-10 (1:2000; 60269-1-1 g; Proteintech, Chicago, IL, USA), anti-Wnt3 (1:1000; ab172612; Abcam) and β-catenin (1:1000; CST8480; Cell Signaling Technology). The anti-tubulin antibody (1:1000; 66031–1-Ig; Proteintech) was used as the loading control.

### CRC xenograft mouse model construction

HCT116 cells (6 × 10^6^ dissolved in 0.1 mL PBS, were subcutaneously injected into the right flanks of BALB/c nude mice. When the tumors became palpable (3–5 mm in diameter), all mice were randomized in a blinded fashion into the following three groups (*n* = 8 per group): Control, IR, and IR+I3A. Mice in the radiotherapy group were subjected to 10-Gy local radiation therapy, which was delivered using an X-RAD 320iX Biological Irradiator (Precision X-ray, North Branford, CT, USA) at a dose rate of 1.0 Gy/min. Tumor volumes were measured with a vernier caliper every 3 days; the following formula was used to calculate tumor volume: V = a*b^2^/2 (a: the longest diameter; b: the shortest diameter). After 3 weeks of irradiation, the mice were sacrificed, and the tumors were isolated. We recorded the weight of each tumor, calculated the average tumor volume, and captured images of tumors in each group.

### Colorectal cancer organoid isolation and culture

Upon arrival, colorectal cancer tumor tissues were photographed and washed in the cold PBS with penicillin/streptomycin (Beyotime, Shanghai, China) for 5 min, and then minced into tiny fragments in a sterile dish on ice. Then tissue fragments were subjected to enzymatic digestion (K601003; BioGenous, Shandong, China) in 10 mL digestion medium containing 9.5 mL basal medium (K601003-A100; BioGenous, Shandong, China) at 37°C for 30–60 min. Tumor organoids were collected after centrifugation at 300 g for 5 min. The resuspended organoids were subsequently mixed with an equal volume of Matrigel® (356231; Corning, NY, USA) and then seeded on a prewarmed 24-well plate at a density of 500 crypts per well. Colorectal cancer organoid complete medium (K2103-CR; BioGenous, Shandong, China) (500 μL) was then added to each well. The organoids were treated with complete medium with or without 100 μM I3A 1 h pre-IR and then exposed to 6 Gy X-rays at a dose rate of 1.1 Gy/min or sham-irradiated. The organoids were finally viewed under an optical microscope (Olympus Corp., Tokyo, Japan), followed by analysis of the organoids using ImageJ software. At least 50 organoids were counted. The study received approval from the Clinical Research Ethics Committee of the Second Affiliated Hospital of Soochow University (Approval Number: JD-LK2023090-I01). Individual informed consent was obtained in writing prior to tissue collection from the patient who contributed to the development of the organoids used in this study. Detailed information about the CRC patient is provided in Supplementary Table S4.

### Statistical analysis

Unless otherwise stated, all statistical analyses and graph plotting were performed using GraphPad Prism 8 (San Diego, CA, USA). Two-tailed Student’s *t* test (parametric) or Mann‒Whitney *U* test (nonparametric) were applied. For comparison of more than three groups, statistical analysis was performed using one-way ANOVA (parametric) or the Kruskal‒Wallis test (nonparametric). *p* < .05 was considered statistically significant.

## Supplementary Material

Supplemental Material

## Data Availability

Research data are available at https://www.ncbi.nlm.nih.gov/bioproject/PRJNA1020984/ and https://www.ncbi.nlm.nih.gov/bioproject/PRJNA1020982/.
