# Supplementary material for: Microbiota-derived I3A protects the intestine against radiation injury by activating AhR/IL-10/Wnt signaling and enhancing the abundance of probiotics
Source: Gut Microbes. 2024 May 5;16(1):2347722. doi: 10.1080/19490976.2024.2347722 (PMC11086037; doi:10.1080/19490976.2024.2347722)
Supplement: Supplemental Material [file KGMI_A_2347722_SM2464.zip › Supplementary Figures clean.docx]

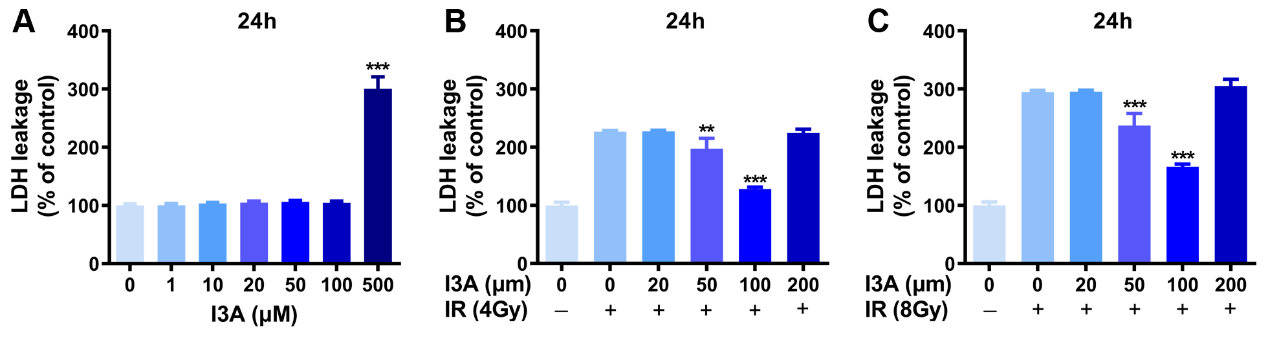


**Supplementary Figure 1.** **Effects of I3A treatment on the cytotoxicity in HIEC-6 cells assessed using the LDH Assay.** (A) Cytotoxicity of I3A in HIEC-6 cells. HIEC-6 cells were treated with the indicated concentrations of I3A for 24 h. Cell death was evaluated using the LDH Cytotoxicity Assay Kit. (B, C) HIEC-6 cells were pretreated with the indicated concentrations of I3A 1 h before 4 or 8 Gy IR, and LDH leakage was measured after 24 h. Data are presented as the mean ± SEM of three independent experiments. ***P* < 0.01; ****P* < 0.001.


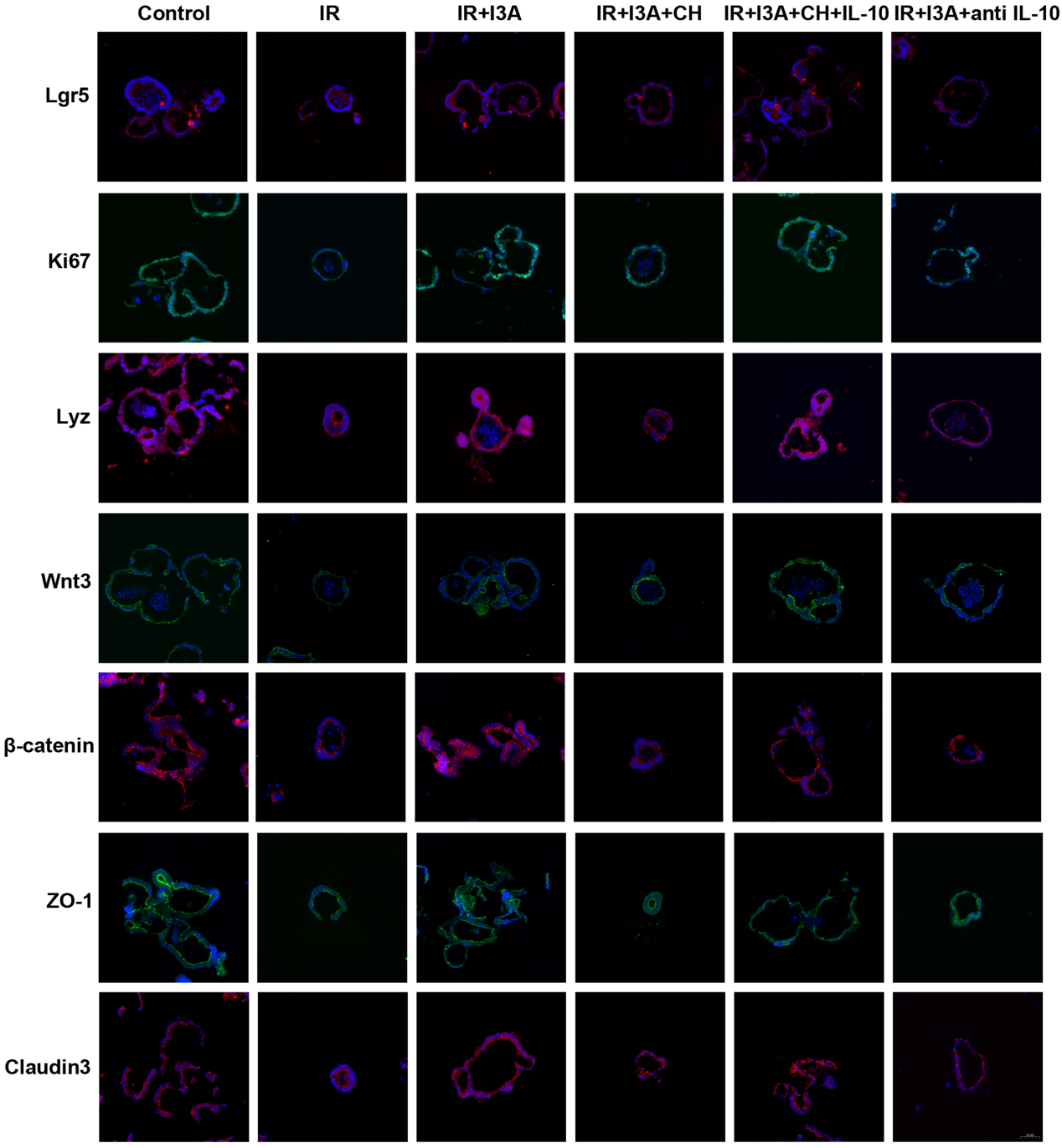


**Supplementary Figure 2. I3A activates the AhR/IL-10/Wnt signaling pathway** **in intestinal organoids.** Representative IF images of intestinal organoids in different groups at 5 d post-IR (Scale bar = 50 μm). Mouse intestinal crypt organoids were treated with vehicle, 100 μM I3A and/or 10 μM CH-223191, 0.1 μg/mL anti-IL-10 and 5 ng/mL IL-10 subjected to 0 or 6 Gy X-ray radiation.
