# Supplementary material for: Microbiota-derived I3A protects the intestine against radiation injury by activating AhR/IL-10/Wnt signaling and enhancing the abundance of probiotics
Source: Gut Microbes. 2024 May 5;16(1):2347722. doi: 10.1080/19490976.2024.2347722 (PMC11086037; doi:10.1080/19490976.2024.2347722)
Supplement: Supplemental Material [file KGMI_A_2347722_SM2464.zip › Table S3 clean.docx]

**Supplementary Table 3.** The primers used in quantitative real-time PCR.

| **Species** | **Gene** | **Forward primer (5′-3′)** | **Reverse primer (5′-3′)** |
| --- | --- | --- | --- |
| Mouse | *CYP1A1* | GACCCTTACAAGTATTTGGTCGT | GGTATCCAGAGCCAGTAACCT |
| Mouse | *KLF11* | CATGGACATTTGTGAGTCGATCC | CCTTTGGTAGATCAGGTGCAG |
| Mouse | *CCNB2* | GCCAAGAGCCATGTGACTATC | CAGAGCTGGTACTTTGGTGTTC |
| Mouse | *FABP6* | CTTCCAGGAGACGTGATTGAAA | CCTCCGAAGTCTGGTGATAGTTG |
| Mouse | *SLC10A2* | GTCTGTCCCCCAAATGCAACT | CACCCCATAGAAAACATCACCA |
| Mouse | *MELTF* | CTGAGCGTGACTTTTTGGCTA | CACAGTGGTCAGCGAGTT |
| Mouse | *β-actin* | GGCTGTATTCCCCTCCATCG | CCAGTTGGTAACAATGCCATGT |
